# Causal AI Recommendation System for Digital Mental Health: Bayesian Decision-Theoretic Analysis

**DOI:** 10.2196/71305

**Published:** 2025-06-19

**Authors:** Mathew Varidel, Victor An, Ian B Hickie, Sally Cripps, Roman Marchant, Jan Scott, Jacob J Crouse, Adam Poulsen, Bridianne O'Dea, Sarah McKenna, Frank Iorfino

**Affiliations:** 1 Brain and Mind Centre, The University of Sydney, 94 Mallett Street, Sydney, 2050, Australia, 61 0293510774; 2 Human Technology Institute, University of Technology Sydney, Sydney, Australia; 3 Institute of Neuroscience, Academic Psychiatry, Newcastle University, Newcastle, United Kingdom; 4 Institute for Mental Health and Wellbeing, Flinders University, Adelaide, Australia

**Keywords:** causal inference, causal structure learning, decision theory, decision analysis, digital health, well-being, psychological distress, functioning, sleep, social support

## Abstract

**Background:**

Digital mental health tools promise to enhance the reach and quality of care. Current tools often recommend content to individuals, typically using generic knowledge-based systems or predictive artificial intelligence (AI). However, predictive AI is problematic for interventional recommendations as cause-effect relationships can be confounded in observed data. Therefore, causal AI is required to compare future outcomes under different interventions.

**Objective:**

We aimed to develop a causal AI recommendation system that uses an individual’s current presentation, their preferences, and the learned dynamics between domains to rank interventions.

**Methods:**

We frame the recommendation problem within a Bayesian decision-theoretic framework, whereby a preference ordering of decisions is estimated using the expected utility of outcomes under interventions. The causal processes are assumed to follow a structural causal model, where the posterior distribution of structural causal models is estimated using a Markov chain Monte Carlo method. Expected utilities under interventions are estimated using a do-operation, which estimates the effects of changing a variable on outcomes, while accounting for confounders. We apply our approach to rank domains relating to mental health and well-being as intervention targets for adults (n=619) who used the Innowell Fitness app between September 2021 to September 2023 and completed a questionnaire at 2 time points (1 wk-6 mo from baseline).

**Results:**

The causal AI recommendation system recommends intervention targets as a function of a user’s baseline presentation, the causal effects of the intervention on itself and other domains, and the utility function. In our example, psychological distress was typically the optimal intervention target in complex cases where multiple domains were unhealthy at baseline, due to it affecting multiple domains with paths to personal functioning (probability [p] of path; p_path_=86%), social support (p_path_=92%), sleep (p_path_=88%), and physical activity (p_path_=86%). The probability of being the optimal intervention target was personal functioning (p_opt_=30%), psychological distress (p_opt_=29%), social support (p_opt_=18%), nutrition (p_opt_=9.6%), substance use (p_opt_=6.7%), sleep (p_opt_=4.5%), and physical activity (p_opt_=2.2%).

**Conclusions:**

This work illustrates the use of causality and decision-theoretic principles to personalize interventions in digital mental health tools.

## Introduction

Theory suggests that mental ill-health and poor well-being are emergent phenomena from the interactions between symptoms within and across domains over time [1-4]. This view recognizes that domains—clinical symptoms (eg, depressive and anxiety symptoms), physical health and activity, social support, personal functioning, sleep, nutrition, alcohol or other substance use—influence each other in complex ways. Furthermore, mental ill-health and well-being are influenced by cultural, socioeconomic, or population-level factors [5,6]. This provides a landscape of potential interventional targets that could alleviate mental ill-health and improve well-being. However, this landscape raises the problem of deciding between interventions, which is made difficult by (1) the heterogeneous presentation of mental ill-health and poor well-being, (2) uncertainty about outcomes under different interventions, (3) timeliness, duration, and sequence of interventions, (4) comparison of multidimensional outcomes given individual-level differences in the prioritization of different symptoms or domains, and (5) costs (monetary or otherwise) associated with interventions.

This interventional decision-making problem can be considered within a Bayesian decision-theoretic (BDT) framework [7]. In BDT, the optimal interventional decision is that which maximizes the expected utility (EU) of outcomes. BDT allows for the incorporation of uncertainties in components of the decision-making process (eg, uncertainty of future outcomes under interventions). Subjective utilities can be used to account for varying individual-level multidimensional outcome preferences and perceived costs. Timing and sequencing can be addressed by conditioning current decisions on prior decisions and observations, while possible future decisions can be marginalized out.

Recommendation systems (RSs) are algorithms that filter content or actions for end users. RSs are often built to filter content to align with an individual’s prior choices or elicited information, and have been successful for this purpose (eg, Netflix [8]). RSs have been applied in digital health in general [9,10], including applications to digital mental health tools [11,12]. RSs in digital mental health have aligned content with user input by resemblance without consideration of outcomes, used expert-level knowledge-based systems typically to align a limited number of inputs with content, or in some cases aligned recommendations with outcomes using predictive artificial intelligence (AI) [13,14]. However, we will aim to build a RS that makes interventional recommendations based on expected outcomes within a BDT framework.

This approach requires methods to predict outcomes under interventions. The use of predictive AI algorithms to align recommendations with outcomes can be problematic as it estimates relationships between inputs and outcomes using potentially confounded conditional probabilities inferred from observational data [15]. As an alternative, we will explore causal AI [16], which includes mechanisms to estimate causal effects from observational data.

**Figure 1.**
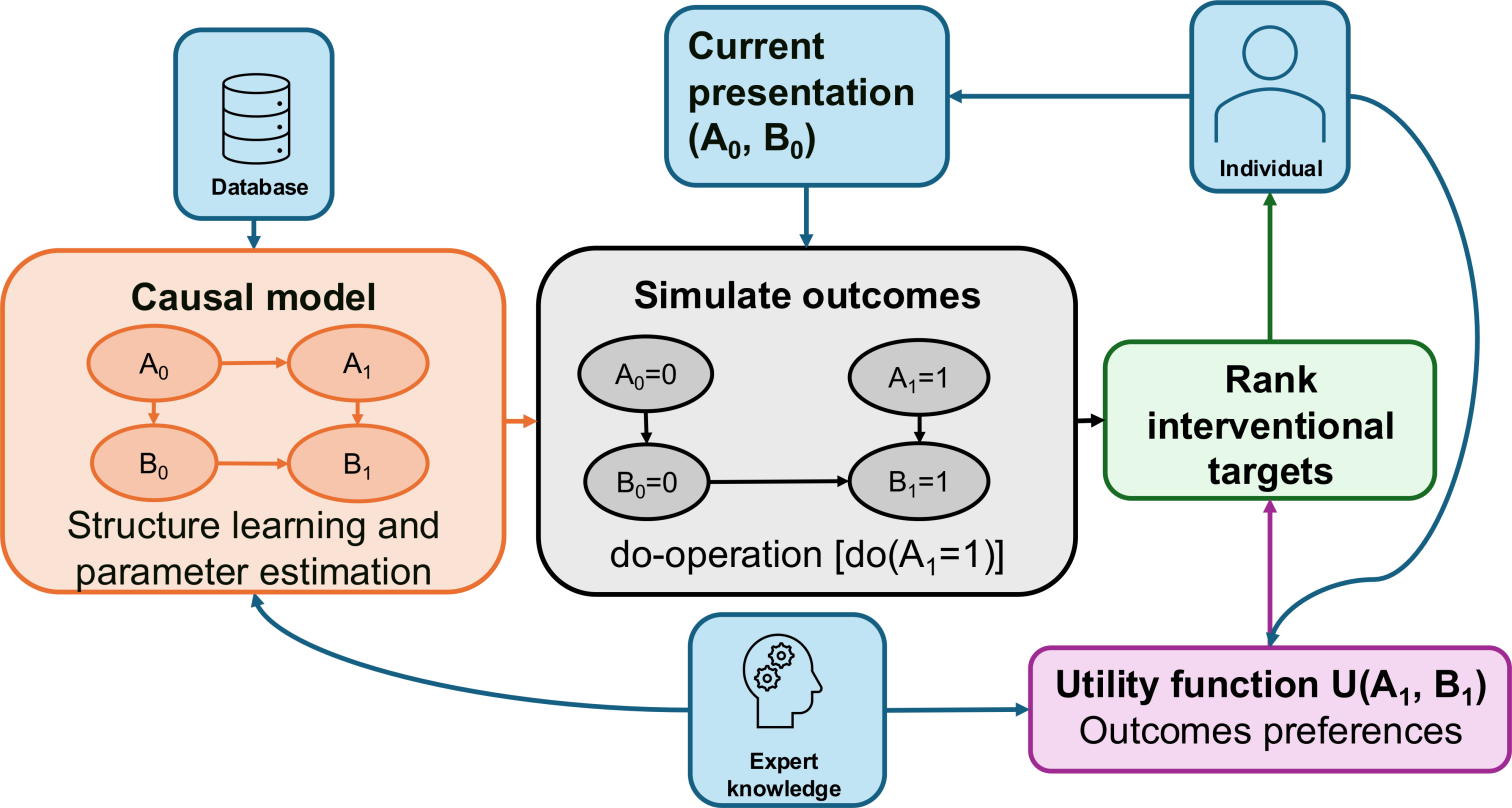
CAIRS uses expert knowledge and observational data to learn a causal model, which is then used to simulate outcomes under idealized interventions and displays intervention targets based on their expected utility. CAIRS: causal artificial intelligence recommendation system.

Many digital technologies aimed at improving mental health and well-being exist today, but their reliance on resemblance, knowledge-based, or predictive AI algorithms limits their ability to provide personalized interventional recommendations [17-19]. While there has been interest in building causal AI applications for decision-support systems within health care generally [20,21], there has been very little work on causal AI applications to digital mental health care. Furthermore, few applications of causal AI have been deployed in digital health in general, as it is a relatively new field that requires further understanding and tools to find real-world applicability. We will outline a causal artificial intelligence recommendation system (CAIRS) that aims to improve interventional decision-making in digital mental health applications (Figure 1). We will then show an application to a general population cohort to rank domains as intervention targets in the mental health and well-being context.

## Methods

### Ethical Considerations

This study analyzed nonidentifiable data collected from the Innowell Fitness app. The dataset contained no personal identifiers or identifying information. In accordance with the National Health and Medical Research Council’s National Statement on Ethical Conduct in Human Research (2023), Section 5.1.17(a), this research was deemed eligible for exemption from ethics review by the University of Sydney Human Research Ethics Committee as it constitutes low risk research using pre-existing, deidentified data. All participants provided consent for their data to be used for this research activity. No participants were compensated for their involvement in this research activity.

### Study Population

This study was conducted using data collected from individuals from an adult population of Australians engaged in work or university education, who were invited to join the Innowell Fitness app through their respective institutions. Individuals within this study used the app between September 2021 to September 2023 (N=5933). Using the Innowell Fitness app, individuals completed a self-report questionnaire that measured their social support, personal functioning, psychological distress, sleep, physical activity, nutrition, and alcohol and other substance use. All items within the questionnaire must be answered to be recorded within the app, and thus, all questionnaires used in this analysis are complete. We used their first completed questionnaire on the app as their baseline observation. As a follow-up observation, we used their first completed questionnaire within a timeframe of 1 week to 6 months postbaseline. Individuals who reported negative minutes spent doing any physical activity were excluded (55/5933, 0.9%). Otherwise, we included all individuals who had at least 1 follow-up from 1 week to 6 months after baseline (619/5878, 11%).

### Innowell Fitness App

The Innowell Fitness app is a digital mental health tool used by adults for the assessment, management, and monitoring of their mental health and well-being [22,23]. It is available on mobile and computer devices, and includes (1) self-report assessments about each domain of a person’s mental fitness, (2) actionable insights about each domain of mental fitness, (3) personalized (knowledge-based) recommendations with evidence-based strategies and resources to understand and manage mental health and well-being, and (4) a goal setting and tracking tool which provides people with habit-forming activities designed to improve their mental fitness. The development of this tool involved a team of psychologists, psychiatrists, mental health research experts, and those with lived experience, who selected items that measure various components of mental health and well-being and collated relevant evidence-based strategies and resources.

The measures and domains assessed in the app include (1) social support, using 3 items from the Schuster Social Support Scale [24]; (2) personal functioning, using 3 items about educational and employment engagement and achievement [25]; (3) psychological distress, using the K6 (Kessler-6) scale for psychological distress [26]; (4) sleep, using 4 sleep items, including feeling refreshed after sleep, trouble falling asleep, and subjective energy [27]; (5) physical activity, 4 items from the International Physical Activity Questionnaire measuring time spent walking, doing moderate exercise, doing vigorous exercise, and being sedentary [28]; (6) alcohol and other substance use, using 3 items about tobacco, alcohol, and other substance use [29,30]; and (7) nutrition, using 2 items about typical composition and portion size of their diet [30]. These individual items are combined to construct domains of interest that are categorized as either “poor,” “fair,” or “healthy.” Item and categorization details can be found in Note S1 in Multimedia Appendix 1.

### Statistical Analysis

Statistical modeling and analyses were performed in R (version 4.3.3; R foundation). Causal inference was performed in the structural causal modeling (SCM) framework. An SCM is described by a set of variables, a set of functions relating the variables, and a causal structure represented by a directed acyclic graph (DAG) that indicates the directionality of causal influence using arrows between random variables. In many causal inference applications, researchers encode causal assumptions within a single SCM, but we suggest that this is not appropriate in the mental health and well-being context, where the causal processes are not well known, and may vary in different contexts or for different individuals. Thus, we will marginalize (ie, Bayesian average) over the distribution of SCMs.

Marginalizing over the SCMs requires the estimation of its posterior distribution. We aimed to sample from the posterior distribution assuming a uniform prior over DAGs, only excluding DAGs with arrows that go backwards in time. Posterior sampling was achieved using an implementation of the Partition Markov Chain Monte Carlo scheme [31,32]. Partition Markov Chain Monte Carlo samples from the space of partitions, where a partition is a set of weakly ordered nodes that represents multiple DAGs. For example, consider a set of variables {A,B,C,D} that have the dependency relations described by the DAG A←B→C→D, then this DAG would be consistent with the partition {{B},{A,C},{D}}. The sampling procedure was run across 8 chains and checked for convergence and resolution (Note S2 in Multimedia Appendix 1). We used the Bayesian Gaussian equivalent score to retain the ordinal information of the random variables.

Simulating outcomes given a DAG is performed by constructing a Bayesian network (BN). A BN assumes nodes take categorical values and relates a variable to its parent variables using conditional probability tables. A BN was constructed per posterior sample by passing the DAG and observed data to the gRain library [33], which estimates the conditional probability tables using a maximum a posteriori estimate. Simulating an outcome given an observed baseline state is performed by setting each baseline node with the values of the observed baseline state and then simulating the follow-up state. This corresponds to doing “nothing” below, which is shorthand for simulating a follow-up state given no intervention. The interventional do-operation is used to simulate outcomes given idealized interventions. This is performed by “mutilating” the BN by removing all edges into an interventional node, setting the interventional node state to “healthy,” and then setting the states of the baseline nodes equal to the given baseline state. Note that this intervention acts on a domain at follow-up.

We assign numerical values to outcomes, which are referred to as utilities in decision theory, to make comparisons between idealized interventions [15,34]. For our primary assumption, we assign the subutility value for outcomes as u=(“poor”=0, “fair”=0.75, “healthy”=1), which corresponds to a moderately risk-averse subutility function. The utility function is then an equal-weighted sum of the subutility values across domains, thus assuming no preference between domains, and ensuring that our focus is on overall well-being. The utility ranges from 0 when all domains are “poor” to 7 when all domains are “healthy.” We use the EU principle to order intervention targets, where we assume that an intervention target *A* is preferred to *B* when the EU for performing an idealized intervention on *A* is greater than on *B*. In our sensitivity analysis, we investigated a risk-neutral subutility function with u=(“poor”=0, “fair”=0.5, “healthy”=1), a risk-averse subutility function u=(“poor”=0, “fair”=1, “healthy”=1), and up-weighting psychological distress and personal functioning compared to all other domains. Further detail about the BDT framework is provided in Note S3 in Multimedia Appendix 1.

We report the average treatment effect (ATE) conditional on the baseline state. This is calculated as the difference in the EU between doing an idealized intervention compared to doing nothing.

## Results

### Sample Characteristics

The sample comprised 619 individuals who used the Innowell Fitness app from the original 5933 cohort. Individuals in our sample tended to have a slightly higher propensity of being in the healthy category across personal functioning (sample n=209, 34%; cohort n=1663, 28%), psychological distress (sample n=326, 53%; cohort n=2818, 48%), nutrition (sample n=268, 43%; cohort n=2388, 40%), physical activity (sample n=466, 75%; cohort n=4284, 72%), sleep (sample n=173, 28%; cohort n=1611, 27%), social support (sample n=180, 29%; cohort n=1714, 29%), and substance use (sample n=383, 62%; cohort n=3471, 59%). The median follow-up time was 55 (IQR 35-96) days, with further breakdown in Note S4 in Multimedia Appendix 1. The sample improved across a range of outcomes from baseline to follow-up where the number of individuals moving from “fair” or “poor” to “healthy” was +45 (7.3%) for sleep, +17 (2.7%) for physical activity, +28 (4.5%) for social support, −1 (0.2%) for personal functioning, +50 (8.1%) for psychological distress, 0 (0%) for substance use, and +24 (3.9%) for nutrition. Further details are provided in Table 1.

**Table 1.** Sample characteristics. Comparison of the analyzed sample (n=619; 10%) to the cohort of working individuals who have used the Innowell Fitness app (N=5933).

		Healthy, n (%)	Fair, n (%)	Poor, n (%)
Sleep				
	Baseline (cohort)	1611 (27)	3282 (55)	1040 (18)
	Baseline	173 (28)	343 (55)	103 (17)
	Follow-up	218 (35)	307 (50)	94 (15)
	Difference between time points	45 (7.3)	−36 (5.8)	−9 (1.5)
Physical activity				
	Baseline (cohort)	4284 (72)	294 (5)	1355 (23)
	Baseline	466 (75)	22 (3.5)	131 (21)
	Follow-up	483 (78)	24 (3.9)	112 (18)
	Difference between time points	17 (2.7)	2 (0.3)	−19 (3.1)
Social support				
	Baseline (cohort)	1714 (29)	1675 (28)	2544 (43)
	Baseline	180 (29)	169 (27)	267 (43)
	Follow-up	208 (34)	205 (33)	203 (32)
	Difference between time points	28 (4.5)	36 (5.8)	−64 (10)
Personal functioning				
	Baseline (cohort)	1663 (28)	1907 (32)	2363 (40)
	Baseline	209 (34)	186 (30)	224 (36)
	Follow-up	208 (34)	181 (29)	230 (37)
	Difference between time points	−1 (0.2)	−5 (0.8)	6 (1)
Psychological distress				
	Baseline (cohort)	2818 (48)	1784 (30)	1331 (22)
	Baseline	326 (53)	171 (28)	122 (20)
	Follow-up	376 (61)	119 (19)	124 (20)
	Difference between time points	50 (8.1)	−52 (8.2)	2 (0.3)
Substance use				
	Baseline (cohort)	3471 (59)	1629 (28)	833 (14)
	Baseline	383 (62)	158 (26)	78 (13)
	Follow-up	383 (62)	158 (26)	78 (13)
	Difference between time points	0 (0)	0 (0)	0 (0)
Nutrition				
	Baseline (cohort)	2388 (40)	2106 (36)	1439 (24)
	Baseline	268 (43)	213 (34)	138 (22)
	Follow-up	292 (47)	206 (33)	121(20)
	Difference between time points	24 (3.9)	−7 (1.1)	−17 (2.7)

### Structure Learning

**Figure 2.**
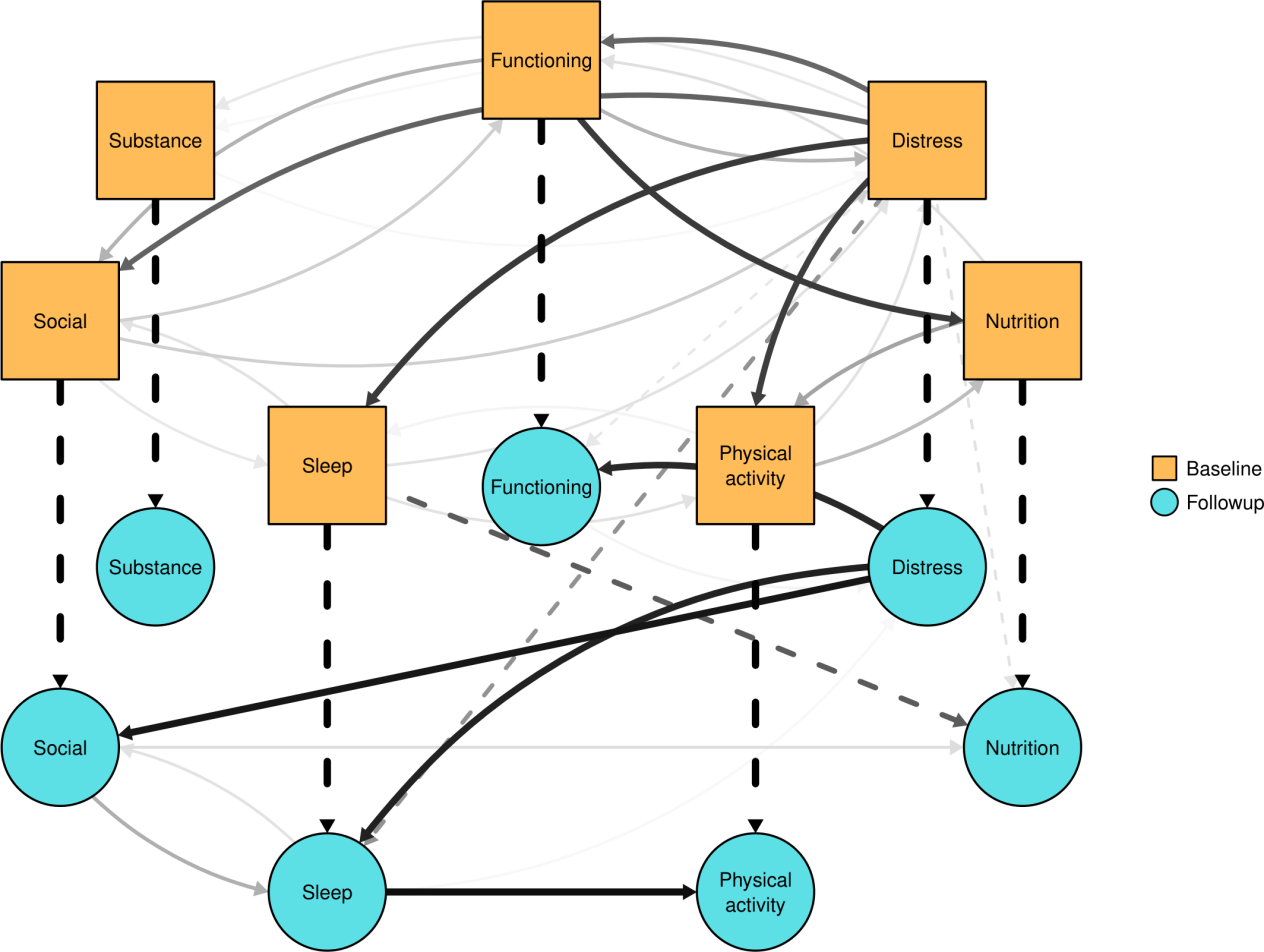
Consensus graph summarizing the posterior distribution of DAGs. Edges between nodes with probability (p; p_parent_>10%), with darker colors corresponding to higher probability. Dashed lines show lagged edges whereas solid lines show within time point edges. DAG: directed acyclic graph.

We now summarize the posterior distribution of DAGs (Figure 2). The contemporaneous network within baseline (ie, $A_{baseline}\to A_{followup}$) shows a dense network consistent with analysis in a partly overlapping sample [22]. There is significant uncertainty about directionality, although psychological distress was more likely to be the parent of another domain than in the opposite direction. Specifically, psychological distress being the parent of (1) personal functioning was 64% compared to 36% in the opposite direction, (2) physical activity was 78% compared to 20%, (3) sleep was 80% compared to 20%, and (4) 65% to social support compared to 27%. Additionally, personal functioning was more likely to affect nutrition (79% compared to 21%).

Adding the time component helps the algorithm differentiate directionality within the follow-up time point. We found the autoregressive edges (ie, $A_{baseline}\to A_{followup}$) had probability (p), p_parent_>99% for all domains. The lagged cross-domain edges (ie, $A_{baseline}\to B_{followup}$) are sleep to nutrition (p_parent_=68%) and potentially psychological distress to sleep (p_parent_=48%). Within the follow-up time point, we found psychological distress to personal functioning (p_parent_=86%), social support (p_parent_=92%), and sleep (p_parent_=88%), along with sleep to physical activity (p_parent_=95%). Including indirect paths, there were paths from psychological distress to personal functioning (p_path_=86%), social support (p_path_=92%), sleep (p_path_=88%), and physical activity (p_path_=86%). Further details are in Note S5 in Multimedia Appendix 1.

### Treatment Effects

**Figure 3.**
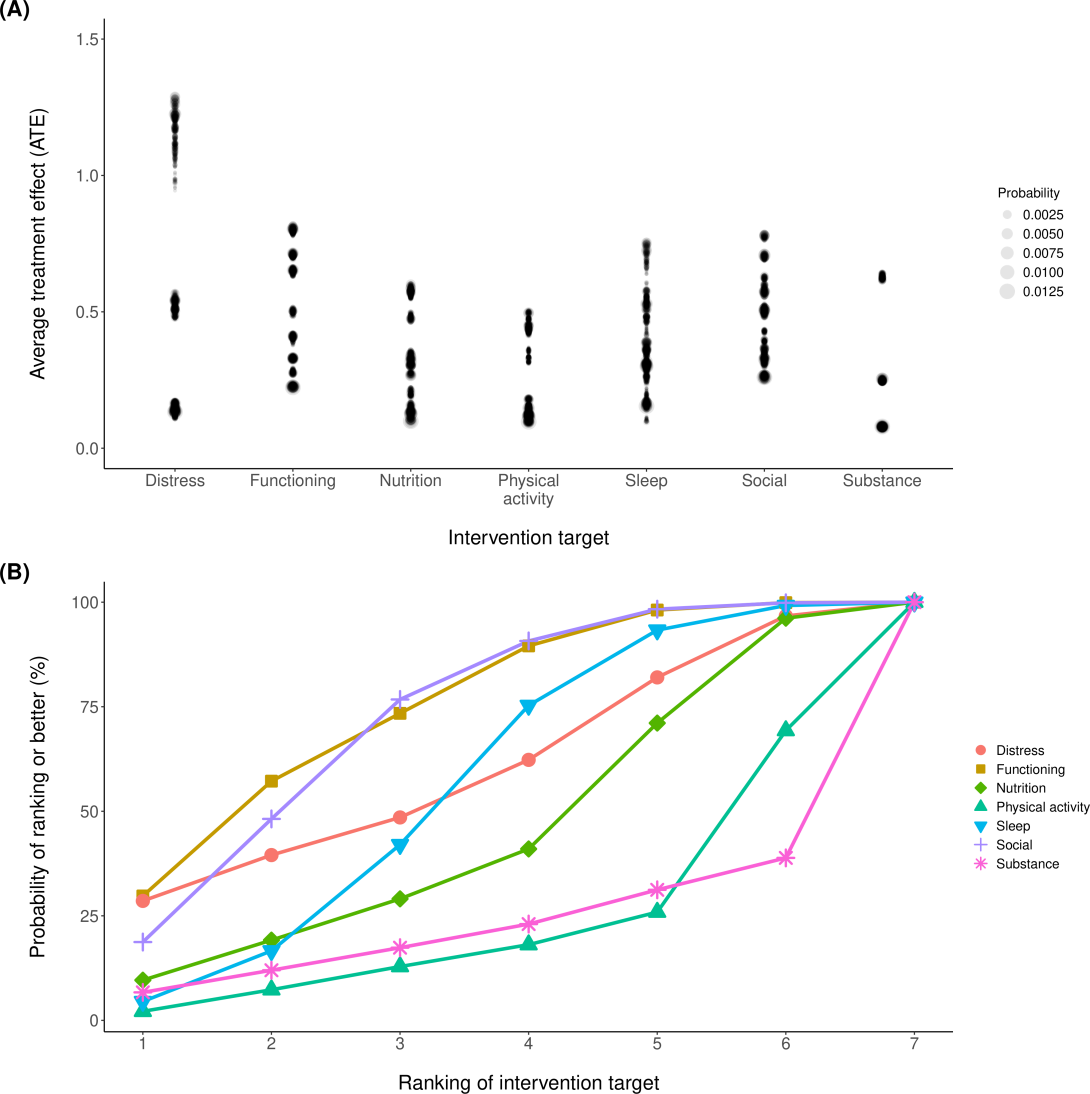
Treatment effects and recommendation targets. Panel (A) shows average treatment effects with point size increasing with an empirical probability of the baseline state. These are converted to recommendations represented in panel (B), where the probability for the rank of each intervention target is marginalized over the baseline states.

Intervening on psychological distress is capable of the greatest ATE, with ATE >1 (equivalent to transitioning from “poor” to “healthy” for 1 domain), when psychological distress itself was adjusted from “poor” to “healthy” while also affecting other domains. Interventions on other domains resulted in ATE less than 1 due to effects being primarily isolated to that domain (Figure 3A).

### Decision Analysis

We estimated a preference ranking for each domain (Figure 3B). The proportion that a domain is the optimal intervention weighted per the probability of the baseline states is personal functioning (p_opt_=30%), psychological distress (p_opt_=29%), social support (p_opt_=18%), nutrition (p_opt_=9.6%), substance use (p_opt_=6.7%), sleep (p_opt_=4.5%), and physical activity (p_opt_=2.2%).

We also report the proportion that a domain would be recommended in a system that presented the top N interventional targets. For example, for a system that reported the top three intervention targets the proportion that each domain would be displayed would be social support (probability of being recommended, p_rec_=77%), personal functioning (p_rec_=73%), psychological distress (p_rec_=49%), sleep (p_rec_=42%), nutrition (p_rec_=29%), substance use (p_rec_=17%), and physical activity (p_rec_=13%).

Optimal interventional targets can be further illuminated by examples. The most common baseline presentation is everything is “healthy,” where the optimal interventional target was social support (EU 6.345, SE 0.034), followed by personal functioning (EU 6.310, SE 0.035), which were greater than doing nothing (EU 6.087, SE 0.033). This is due to a regression to the mean effect, where social support (expected subutility [Eu] 0.764, SE 0.010) and personal functioning (Eu 0.784, SE 0.010) tend to revert to unhealthy states with greater probability than other domains, which all had Eu >0.9.

Domains that are less healthy than all other domains are typically the optimal interventional target. However, when multiple domains including psychological distress are “fair” with either nutrition or physical activity as “poor,” psychological distress was the optimal target. For example, psychological distress is the optimal intervention target for the state (functioning=“fair,” psychological distress=“fair,” nutrition=“fair,” physical activity=“poor,” sleep=“fair,” social=“fair,” substance=“healthy”), as it affects multiple domains.

**Figure 4.**
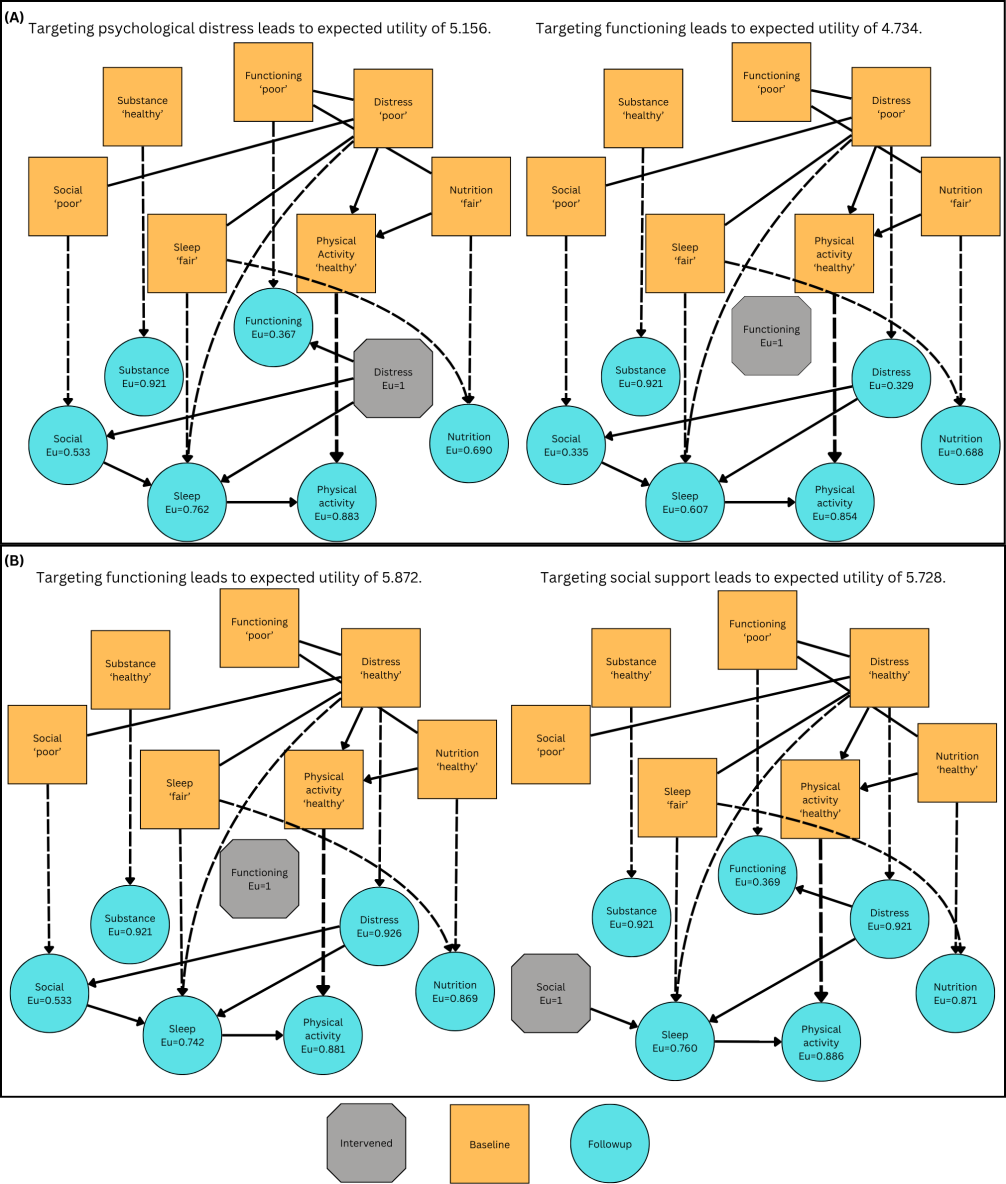
Comparison of predicted outcomes and utilities following interventions for different baseline presentations shown in panel (A) and (B). We show edges in the maximum a posteriori completed partially DAG, where undirected edges correspond to cases where DAGs with edges in either direction have the same posterior probability. Dashed lines show lagged edges whereas solid lines show within time point edges. DAG: directed acyclic graph; Eu: expected subutility.

Psychological distress was the optimal intervention if it was equally unhealthy to any other domain. For example, assuming the baseline state (functioning=“poor,” psychological distress=“poor,” nutrition=“fair,” physical activity=“healthy,” sleep=“fair,” social=“poor,” substance=“healthy”), the optimal intervention target is psychological distress (EU 5.156, SE 0.115), rather than personal functioning (EU 4.734, SE 0.087), or social support (EU 4.638, SE 0.122) despite those domains also being poor (Figure 4A).

When psychological distress was “healthy” and multiple domains were unhealthy, the interventional target was less certain. For example, for the state (functioning=“poor,” psychological distress=“healthy,” nutrition=“healthy,” physical activity=“healthy,” sleep=“fair,” social=“poor,” substance=“healthy”), the optimal interventional target was personal functioning (EU 5.872, SE 0.059) instead of social support (EU 5.728, SE 0.067). This is for similar reasons that the unhealthy personal functioning state persists with greater probability than social support (Figure 4B).

We tested the sensitivity of preference rankings to utility function assumptions. These different assumptions could represent different expert or individual-level outcome preferences. Assuming a risk-neutral subutility function with u=(“poor”=0, “fair”=0.5, “healthy”=1), the optimal intervention targets were psychological distress (p_opt_=28%), personal functioning (p_opt_=27%), social support (p_opt_=18%), sleep (p_opt_=11%), nutrition (p_opt_=7.4%), substance use (p_opt_=7.1%), and physical activity (p_opt_=1.4%). We then tested for a highly risk-averse subutility function u=(“poor”=0, “fair”=1, “healthy”=1), where we found psychological distress (p_opt_=28%), personal functioning (p_opt_=28%), social support (p_opt_=24%), nutrition (p_opt_=8.2%), substance use (p_opt_=6.7%), physical activity (p_opt_=4.3%), and sleep (p_opt_=0.9%). We also increased the weighting of psychological distress and personal functioning using weakly ordered domain-ranking preferences, where we found distress (p_opt_=39%), personal functioning (p_opt_=37%), social support (p_opt_=11%), nutrition (p_opt_=5.7%), substance use (p_opt_=4.3%), sleep (p_opt_=1.9%), and physical activity (p_opt_=1.2%). Further details are in Note S6 in Multimedia Appendix 1.

## Discussion

### Principal Findings

We developed a CAIRS that can be incorporated into digital mental health care tools. Our primary contribution is to show how causal effects and their uncertainty, estimated using causal AI, can be incorporated into automated RSs. We also explored how those recommendations would change per expert or individual-level outcome preferences.

Using our example, we found the optimal interventional target is a function of an individual’s presentation (which in our example is their baseline state), the noninterventional transition from baseline to follow-up, the causal effects of the intervention on itself and other domains, and the utility function. To summarize our results, the optimal interventional target assuming the default utility function was (1) the unhealthiest baseline domain with some exceptions where psychological distress is more effective than intervening on “poor” nutrition or physical activity, (2) psychological distress when it is equally or more unhealthy than other domains, or (3) the domain that is more likely to transition to or persist in an unhealthy state.

### Personalizing Recommendations

CAIRS provides mechanisms to personalize recommendations. In our example, conditioning recommendations on an individual’s baseline presentation leads to recommending interventions consistent with an individual’s current or predicted future problem domains. Intervening on the unhealthiest domain would be the conclusion of most systems. Similarly, domains that are more likely to persist in or transition to unhealthy states suggest that intervention is required. While many RSs may implement this latter step if known, we note that this finding was not something we considered before performing the analysis and is not incorporated into the current Innowell Fitness knowledge-based recommendations, suggesting added value from our analysis.

We also explored personalization of recommendations to an individual’s outcome preferences. Comparing multidimensional outcomes per expert or individual preferences is a challenging task that BDT provides a framework to computationally encode within an RS. Some possibilities of varying outcome preferences were explored by adjusting the subutility function to account for different risk-aversity and changing the domain weightings, which corresponded to slight recommendation changes. Further adjustments in the utility function are possible, and this utility framework will become increasingly important as we increase the number of domains or investigate symptoms, as it is unlikely that all relevant domains or symptoms would be considered equally important by all individuals or experts.

Incorporating personalized outcome preferences in a deployed app would require a utility elicitation mechanism. There are many utility elicitation methods available, some of which are not overly burdensome, such as eliciting weakly ordered preference for domains [35], while BDT provides mechanisms to stop utility elicitation when there is enough information to make a recommendation [36]. With that said, our analysis does not address the feasibility of eliciting individualized utilities in real-world applications, which should be considered for further research. Further preferences (eg, associated costs or interventional preferences) would also improve the personalization of the recommendations.

BDT could find applicability elsewhere within digital health applications. It could also be applied to other causal inference or predictive frameworks, such as undirected or dynamic networks, where intervention targets have been explored [37-42], but often assume that all domains or symptoms are equally important and typically do not marginalize out uncertainties. We may also want to balance mental health and well-being outcomes with other considerations, such as user engagement, which is often poor in digital mental health tools [43-45]. We keep this out of the current scope, only noting that utility functions are a broad framework that can be used to incorporate a range of aspects for optimization.

### Recommending Interventions

Within the clinical setting, CAIRS will aid the implementation of highly personalized care [4]. In these frameworks, the effects between multiple dimensions are considered to inform the processes that affect an individual’s mental ill-health and improve interventional decision-making. These approaches are often supplemented by ongoing measurement-based care to monitor an individual’s progress [46], with several digital mental health tools that support implementation [23]. While these tools have aided highly personalized care across domains, they rarely provide multidimensional interventional insights beyond simple knowledge-based approaches. CAIRS could enhance these tools by providing a data-driven understanding of the causal processes between domains, along with simplifying decisions by filtering interventions or intervention targets across several domains.

Furthermore, in cases where interventions are well recorded, it would be possible to include them in the SCMs, such that they could be recommended algorithmically. Although, causal discovery with interventions can be difficult as they often influence multiple variables [47] and the ethical challenges of recommending interventions will need to be considered. Including clinical symptoms and other relevant clinical variables would also improve the specification of interventions. Additionally, the SCMs will need to be sampled within each new setting.

An advantage of our approach is the improved explainability of the recommendations compared to most AI algorithms. Causal insight can be communicated to clinicians or individuals by displaying the SCMs graphically, similar to what is shown in Figure 4. Training would be required for clinicians to fully understand the outputs of CAIRS. However, clinicians undergo causal reasoning during case formulation similar to that in CAIRS [48], and thus, we suspect it will be feasible for clinicians to understand its recommendations and insights.

In our example, targeting psychological distress should be preferred over other equally unhealthy domains in this general population cohort of adults engaged in the workforce or university education. This result is due to the causal effects that psychological distress has on personal functioning, social support, sleep, and physical activity (mediated by sleep). This is consistent with current understanding that mental ill-health affects multiple domains, including work concerning absenteeism and productivity [49,50], sleeping patterns, for example, due to rumination [51,52], and social connection due to social anxiety [53].

This has interventional implications for individuals within this cohort. Psychological distress as measured using the K6 scale has been shown to have very good accuracy at identifying serious mental ill-health in general populations, typically defined as any mental disorder meeting diagnostic threshold within the following 12 months [54,55]. Therefore, CAIRS could identify and direct individuals from this general population to targeted clinical interventions, such as cognitive behavioral therapies, which have been shown to be efficacious for a range of serious mental illnesses within many contexts [56], including internet-delivered therapies [57,58]. The advantage of CAIRS rather than solely using a K6 score is to balance psychological distress against other considerations, including other problem domains and any other individualized preferences or costs. Thus, CAIRS could reduce clinical resources in the assignment of individuals to relevant therapies, which could lead to improved access to such therapies.

### Comparisons to Prior Work

RSs within digital mental health and well-being contexts have been used to recommend content to individuals [13,14]. These algorithms recommend content to individuals using the resemblance of user input, expert-level knowledge-based systems, or predictive AI. While these algorithms may be useful in some contexts, they do not appropriately consider outcomes under interventions, which we argue is fundamental to recommending interventions, and is incorporated in CAIRS.

Components of our approach have been explored previously. Causal AI with structure learning has been used for epidemiological purposes in mental health [59-61]. These analyses have found a wide array of dependencies between variables, both cross-sectionally and longitudinally. Those analyses have been used to estimate average causal effects using a counterfactual query of the SCM, which provides a broad understanding of interventional targets. Our approach improves on this by leveraging personalized information, including their current state and outcome preferences, which is required to make individual-level decisions.

BDT principles have also been applied for health care decision problems [62-64]. These applications typically assume a causal structure (often implicitly rather than explicitly through a DAG), which may not be appropriate for applications in mental health and well-being, where the causal structure may vary. Therefore, combining causal structure learning and BDT to account for the uncertainty in the SCM provides a novel approach for applications in digital mental health.

### Analytical Limitations

First, our results rely on causal interpretations of the inferred DAGs. Assumptions must hold for this to be true, including that we have all relevant confounders and colliders [65]. Missing variables can lead to incorrectly inferred SCMs, which would lead to incorrect causal effects and recommendations. For the causal effect estimations to be valid other factors such as sociodemographics, historical values before baseline, or other domains have a negligible causal effect on follow-up variables beyond the effect that they have on the observed variables. In our example, a wide array of domains that could feasibly interact were investigated, and thus, this assumption may be reasonable. However, these assumptions may not hold and should be tested more thoroughly, including improving the recording of potential confounders within the Innowell Fitness app.

Second, transferability of our results from our example to other cohorts is also limited. The relevant variables should be tailored to that cohort and the SCMs will have to be learned within that context. With that said, we suspect that the CAIRS approach will be broadly applicable to a wide array of contexts.

Third, we acknowledge that a data-driven approach to causal discovery, as we have attempted, may not be adequate to accurately estimate SCMs. Although we suspect that the full SCM is unlikely to be well known in the mental health and well-being context, some aspects of the causal structure may be known, or at least highly probable, given knowledge about those constructs within a specific context or for an individual. Bayesian inference for causal structures allows for this prior knowledge to be incorporated into the structure learning procedure [16]. Therefore, future iterations of CAIRS should incorporate prior causal knowledge into the procedure.

Fourth, the true causal paths are probably more complex than suggested in this work, as more causal effects have been found in other contexts between the domains that we have studied. The data is likely underpowered to determine all causal paths. However, the BDT framework used within this analysis accounts for uncertain variables, including the SCM, which should allow for appropriate recommendations given the information available in the data.

Lastly, within time point, cyclic paths will be missing due to the acyclic constraint on the causal structure. The acyclic limitation reduces the cross-domain causal effects within a given time point, which may underestimate the effects of interventions in some cases. Accounting for this appropriately will require the incorporation of recent methodological developments [42], which allow for alternative representations of SCMs to account for cycles.

### Conclusions

Interventional recommendation algorithms require careful incorporation of causal considerations and decision-making principles. Important aspects are the estimation of outcomes under interventions requiring causal modeling, the assignment of appropriate utilities to align recommendations with outcome preferences, and consideration of uncertainty. These considerations can be incorporated into computational models using causal AI and BDT. In our example application aimed at a general population, we show that CAIRS can recommend reasonable intervention targets across multiple domains, which we argue could be used to assign intervention targets, such as directing individuals to clinical therapies. Therefore, this approach has the potential to streamline recommendations for complex decisions that are common in digital mental health care.

## Supplementary material

10.2196/71305 Multimedia Appendix 1 Additional information and results.

## Abbreviations

AI: artificial intelligence
ATE: average treatment effect
BDT: Bayesian decision-theoretic
BN: Bayesian network
CAIRS: causal artificial intelligence recommendation system
DAG: directed acyclic graph
EU: expected utility
Eu: expected subutility
K6: Kessler-6
RS: recommendation system
SCM: structural causal model
